# Design of N-Terminal Derivatives from a Novel Dermaseptin Exhibiting Broad-Spectrum Antimicrobial Activity against Isolates from Cystic Fibrosis Patients

**DOI:** 10.3390/biom9110646

**Published:** 2019-10-24

**Authors:** Yuan Ying, Hui Wang, Xinping Xi, Chengbang Ma, Yue Liu, Mei Zhou, Qiang Du, James F. Burrows, Minjie Wei, Tianbao Chen, Lei Wang

**Affiliations:** 1 School of Pharmacy, China Medical University, Shenyang 110001, China; yying01@qub.ac.uk (Y.Y.); hwang@cmu.edu.cn (H.W.); mjwei@hotmail.com (M.W.); 2 Natural Drug Discovery Group, School of Pharmacy, Queen’s University Belfast, Belfast BT9 7BL, Northern Ireland, UK; c.ma@qub.ac.uk (C.M.); yliu58@qub.ac.uk (Y.L.); m.zhou@qub.ac.uk (M.Z.); j.burrows@qub.ac.uk (J.F.B.); t.chen@qub.ac.uk (T.C.); l.wang@qub.ac.uk (L.W.)

**Keywords:** antimicrobial peptide, dermaseptin, peptide design, cystic fibrosis infection

## Abstract

Dermaseptins are an antimicrobial peptide family widely identified from the skin secretions of phyllomeudusinae frogs. Here, we identify Dermaseptin-PC (DM-PC), from the skin secretion of *Phyllomedusa coelestis*, and further investigate the properties of this peptide, and a number of rationally designed truncated derivatives. The truncated 19-mer derived from the N-terminus exhibited similar antimicrobial potency when compared to the parent peptide, but the haemolytic effect of this truncated peptide was significantly decreased. Based on previous studies, the charge and hydrophobicity of truncated derivatives can affect the bioactivity of these peptides and thus we designed a 10-mer derivative with an optimised positive charge and a cyclohexylalanine (Cha) at the C-terminus for enhancing the hydrophobicity, DMPC-10A, which retained the antimicrobial activity of the parent peptide. To further investigate the influence of Cha at the C-terminus on activity, it was substituted by alanine (Ala) to generate another derivative, DMPC-10, but this was found to be much less potent. In addition, DM-PC, DMPC-19 and DMPC-10A not only rapidly killed planktonic bacteria isolated from cystic fibrosis (CF) patient, but also effectively eradicated their biofilm matrices.

## 1. Introduction

Antimicrobial peptides (AMPs) form part of the innate immune response, of many organisms, including bacteria, fungi, amphibians, insects, mammals, plants and many others [1]. So far, hundreds of naturally occurring AMPs have been isolated and characterised as highly efficacious, safe, and tolerable antimicrobials [2,3]. Also, given their ability to interact with bacterial cytoplasmic membranes in a manner not dependent upon specific receptors, AMPs also have potential as broad-spectrum antimicrobials [4,5] and advances in our understanding of their modes of action, have given us new insights into their potential for development as novel therapies to treat multidrug-resistant bacterial infections [6,7].

The chronic inflammation in cystic fibrosis (CF) patients represent the strong mucus phenotype of which provides a pleasant environment for bacteria to obtain antibiotic resistance in biofilms. To be specific, MRSA and *P. aeruginosa*, the prevalent infectious human pathogens, colonising the respiratory tract and responsible for severe infection in people with CF [8]. In addition, *P. aeruginosa* permanently colonises cystic fibrosis lungs despite the aggressive use of antibiotics, which employs some strategies that promote chronic pulmonary colonisation instead of acute infection [9]. Whilst, AMPs have demonstrated outstanding efficacy against these strains, which is a promising therapeutic approach to overcome the drug-resistant strains isolated from cystic fibrosis (CF) patients [10,11].

Dermaseptins, which are linear polycationic (lysine-rich) AMPs, are found in the skin of *Phyllomedusa* frogs [12]. Generally, they are composed of 28–34 amino acids and structured in amphipathic helices in nonpolar solvents, which means these compounds undergo coil-to-helix transition upon binding to lipid bilayers [13]. Despite sequence similarities, the dermaseptins, and their analogues, differ in their potency and spectrum of antimicrobial activities [14]. Overall, they tend to cause rapid lysis of a range of microorganisms in vitro, whilst they have limited or no toxic effect on mammalian cells. Structure-activity relationship studies of dermaseptin-derived peptides have led to the identification of truncated mimetics. In particular, Feder et al. demonstrated that the prerequisite for the antimicrobial activity of dermaseptins is the N-terminal helical domain rather than the C-terminal tail [15,16]. With the identification and modification of the topological domains that are responsible for bioactivity, their selectivity between bacteria and mammalian cells can be completely reversed by optimising the length and hydrophobicity of C-terminal domain of dermaseptins [17].

In this study, a novel dermaseptin, named DM-PC, was identified from the skin secretion of *Phyllomedusa coelestis* by molecular cloning and MS/MS sequencing. DM-PC showed a non-specific bactericidal effect but was observed to possess moderate haemolytic activity. Two truncated derivatives DMPC-19 and DMPC-10A were designed and they maintained potent and broad-spectrum antimicrobial activity against a panel of laboratory reference and clinically isolated Gram-positive and Gram-negative bacterial strains from CF patients.

## 2. Materials and Methods 

### 2.1. Acquisition of Skin Secretion of South American Tree-Frog, Phyllomedusa Coelestis

Specimens of *Phyllomedusa coelestis* (*n* = 3) were obtained from commercial sources in the United States. The skin secretion was acquired by mild transdermal electrical stimulation along with hand massaging of the dorsal skin [18]. Harvested secretion was dissolved in distilled deionised water (ddH_2_O), snap-frozen by liquid nitrogen prior to lyophilisation, and preserved at −20 °C before analysis. The animal study was conducted in China Medical University that was approval by the China Medical University Ethical Review Board with an approval code: 2019–052.

### 2.2. Identification of DM-PC Precursor-Encoding cDNA from Skin Secretion

The mRNA from skin secretion was isolated and skin secretion-derived cDNA library of *Phyllomedusa coelestis* was constructed as described previously [19]. The degenerate sense primer (5′-CCMRWCATGKCTTTCHTDAAGAAATCT-3′) was designed from a highly-conserved domain within the 5′-untranslated regions and signal peptide domains of closely-related *Phyllomedusa* species. The cDNA was amplified via RACE techniques and the purified products were cloned by pGEM-T easy vector system (Promega, Madison, W1, USA). Finally, each clone was sequenced by ABI 3100 automated capillary sequencer (Applied Biosystems, Foster City, CA, USA) using BigDye Terminator v3.1 Cycle Sequencing Kit (Life Technologies, Paisley, UK).

### 2.3. Isolation of the Putative Mature Peptide from Skin Secretion

The isolation and identification of mature peptides in skin secretion were performed as in a previous study [19]. In brief, peptides in chromatographic fractions with identical molecular mass to that calculated for the putative peptide were subjected to tandem mass spectrometry fragmentation sequencing. The tandem mass spectra were subjected to Thermo Scientific Proteome Discoverer 1.0 software, via Sequest algorithm against the self-defined Fasta database (Thermo Fisher Scientific, San Jose, CA, USA).

### 2.4. Peptide Synthesis

The natural peptide discovered, as well as its analogues, were chemically-synthesised using a Tribute Peptide Synthesiser (Protein Technologies, Tucson, AZ, USA). Peptides were synthesised from C-terminal end to N-terminal end as previous study [17]. The Fmoc protecting groups were deprotected using 20% (*v*/*v*) piperidine in DMF, and each amino acid residue was activated and coupled using 11% (*v*/*v*) NMM in 89% (*v*/*v*) DMF combined with activator HBTU. The synthetic peptides were purified by RP-HPLC and lyophilized for functional tests.

### 2.5. Secondary Structure Analysis of Synthetic Peptide

Helical wheel plots and physiochemical properties of peptides were obtained from Heliquest. In addition, circular dichroism (CD) analysis was conducted using a JASCO J815 Spectropolarimeter (JASCO Inc., Jasco, Essex, UK), which was performed in our previous study [17]. Generally, peptides were dissolved in either 10 mM ammonium acetate buffer or 50% TFE in 10 mM ammonium acetate buffer at a concentration of 100 μM, and placed in a 1-mm quartz cuvette. The scan range was subjected to 190–250 nm with 0.5 nm data pitch, at 100 nm/min scanning speed. The spectra were obtained by averaging data from three scans at 20 °C.

### 2.6. Antimicrobial Assays

The antimicrobial activity of each synthetic peptide was evaluated by the standard microdilution assay as described previously [19], with minor modifications. Briefly, a panel of reference (*S. aureus* NCTC 10788, *E. coli* NCTC 10418, MRSA ATCC 12493, *E. faecalis* NCTC 12697, *K. pneumoniae* ATCC 43816, *P. aeruginosa* ATCC 27853 and *C. albicans* NCYC 1467) and clinically isolated bacteria (MRSA B038 V1S1 A, MRSA B042 V2E1 A and *P. aeruginosa* B004 V2S2 B) were employed for the determination of minimum inhibitory concentration (MIC). The minimum bactericidal concentration (MBC) was detected by sub-culturing treated samples onto Mueller-Hinton agar (MHA). Furthermore, the antimicrobial activity in the presence of 2, 5, 10 mM of MgCl_2_ was investigated. All tests were performed with a peptide concentration range from 256-1 µM in two-fold dilution and the assays were done by triplicate.

### 2.7. Time Killing Assay

The instantaneous viable cell count was evaluated according to the method adopted by Khan et al. [20]. In brief, mid-log phase bacterial culture (10^7^ CFU/mL) were inoculated into culture medium MHB containing peptides at the concentration of 8 µM and incubated at 37 °C with shaking (200 rpm). Appropriate time points were selected, as indicated, to observe the viable counts.

### 2.8. Membrane Permeability Kinetic Assay

SYTOX™ Green Nucleic Acid Stain (Thermo Fisher Scientific, Waltham, MA, USA), a fluorescent dye that is completely excluded from living cells, was used to assess the integrity of the plasma membranes of bacteria [21]. Briefly, peptides at the concentration of 8 µM were mixed with bacteria suspension in a 96-well black plate, followed by staining with SYTOX^TM^ Green immediately. Then, the fluorescent intensity was measured with a Synergy HT plate reader (BioTek, Minneapolis, MN, USA) for 60 min (interval 5 min) without incubation by an excitation and emission wavelength of 485 and 528 nm, respectively.

### 2.9. Minimal Biofilm Inhibitory Concentration (MBIC) and Minimal Biofilm Eradication Concentration (MBEC) Assays

MBIC and MBEC assays were performed using 96-well microplates, and biofilm mass was evaluated by crystal violet staining assay. The clinical isolates, MRSA (B038 V1S1 A), MRSA (B042 V2E1 A) and *P. aeruginosa* (B004 V2S2 B) from cystic fibrosis (CF) patients, were employed in this study as our previous study [9]. Gram-positive bacteria were inoculated overnight in tryptic soy broth (TSB), while the Gram-negative bacteria formed in LB. Briefly, for MBIC assay, broth-diluted bacteria (5 × 10^5^ CFU/mL) was treated with different peptide solutions from 1 to 256 µM at 37 °C for 24 h. For MBEC assay, a period of 48 h incubation was maintained to achieve the formation of biofilm, then the PBS-washed biofilm was treated with peptide solutions at 37 °C for 24 h. The plates were washed with PBS, fixed with methanol for 10 min, stained by 125 μL 0.1% crystal violet solution for 30 min (Sigma-Aldrich, Gillingham, UK). Dissolved crystal violet in each well by 30% acetic acid (Sigma-Aldrich, Gillingham, UK) was recorded by Synergy HT plate reader at 595 nm.

### 2.10. Haemolysis Assay

The haemolytic activity of synthetic peptides was assessed as our previous study [9], using the defibrinated horse red blood cells. Briefly, a 2% red blood cell suspension was treated with different peptide concentrations from 256 µM to 1 µM at 37 °C for 2 h. 1% Triton-X 100 was employed as positive controls. 100 μL of the supernatant from each sample was transferred to a microtiter plate after incubation and the absorbance was measured with a Synergy HT plate reader at 550 nm (BioTek, Minneapolis, MN, USA).

## 3. Results

### 3.1. Identification and Characterisation of DM-PC from the Skin Secretion

**Figure 1:**
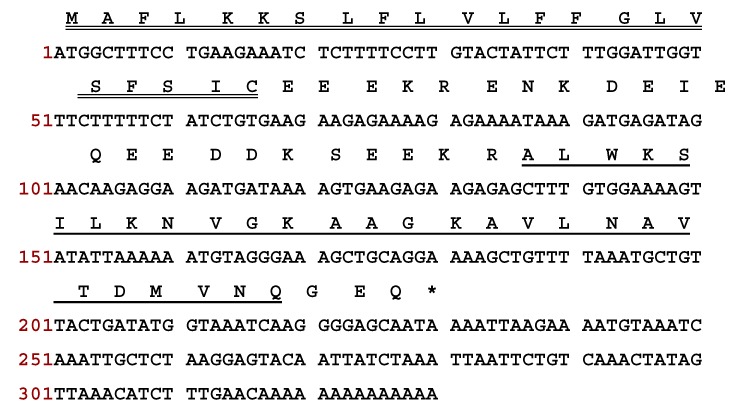
Nucleotides and translated open-reading frame amino acid sequences of a cloned cDNA encoding the biosynthetic precursor of DM-PC. The putative signal peptide sequence is double-underlined, while the putative mature peptide sequence is single-underlined, and the stop codon is indicated by an asterisk.

**Figure 2:**
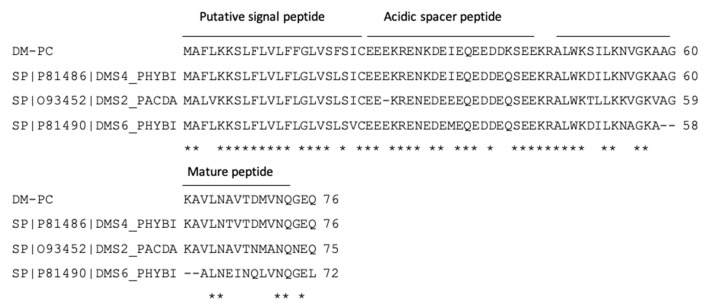
Alignments of cDNA-deduced open-reading frame amino acid sequences of DM-PC and top NCBI BLAST analytes. Fully conserved residues are marked by asterisks.

A novel bioactive peptide precursor-encoding cDNA was consistently cloned from the *Phyllomedusa coelestis* skin secretion-derived cDNA library. The novel gene-encoded peptide was named DM-PC (Figure 1). As shown in Figure 2, DM-PC demonstrates a similar topological structure compared to other dermaseptin peptides, including a highly conserved putative signal peptide, an acidic amino acid residue-rich ‘spacer’ peptide region and a mature peptide region following a typical Lys-Arg- (-KR-) propeptide convertase processing site. The C-terminal glycine residue acts as an amide donor to terminate the glutamine residue of the mature peptide and results in post-translational amide modification (Sequence deposited to Genbank; accession No. MN431956).

**Figure 3:**
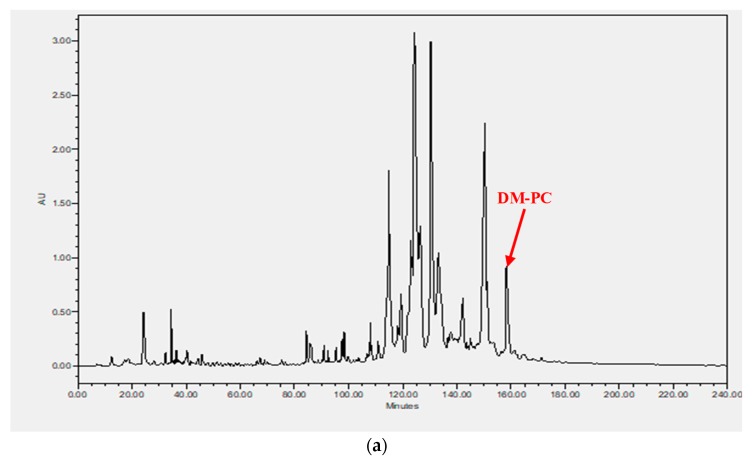

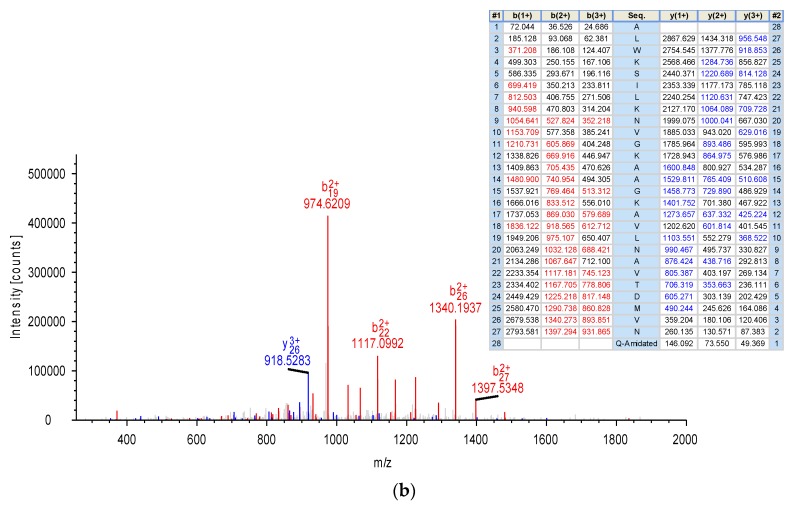
Identification of DM-PC derived from the skin secretion of *P. coelestis* (**a**) RP-HPLC chromatogram of skin secretion of *P. coelestis* monitored at 214 nm. The red arrow indicates the retention time of DM-PC. (**b**) Annotated MS/MS spectrum of DM-PC. b- and y-ions arising from collision induced dissociation of the triply charged precursor ion (980.30 *m*/*z*, [M + 3H]^3+^) are observed and indicated in the table in blue and red typeface.

*Phyllomedusa coelestis* skin secretion was fractionated by RP-HPLC (Figure 3a) and the fractions were further analysed by MS/MS against the custom database (Figure 3b). It revealed that DM-PC was eluted at 159 min. The Sequest mapping data listed the observed b and y fragments generated from DM-PC and the presence of b_27_^2+^ ion confirmed that the Gly residue at the end of the peptide precursor contributed to the post-translational modification of C-terminal amidation for DM-PC.

### 3.2. Peptide Design

**Figure 4:**
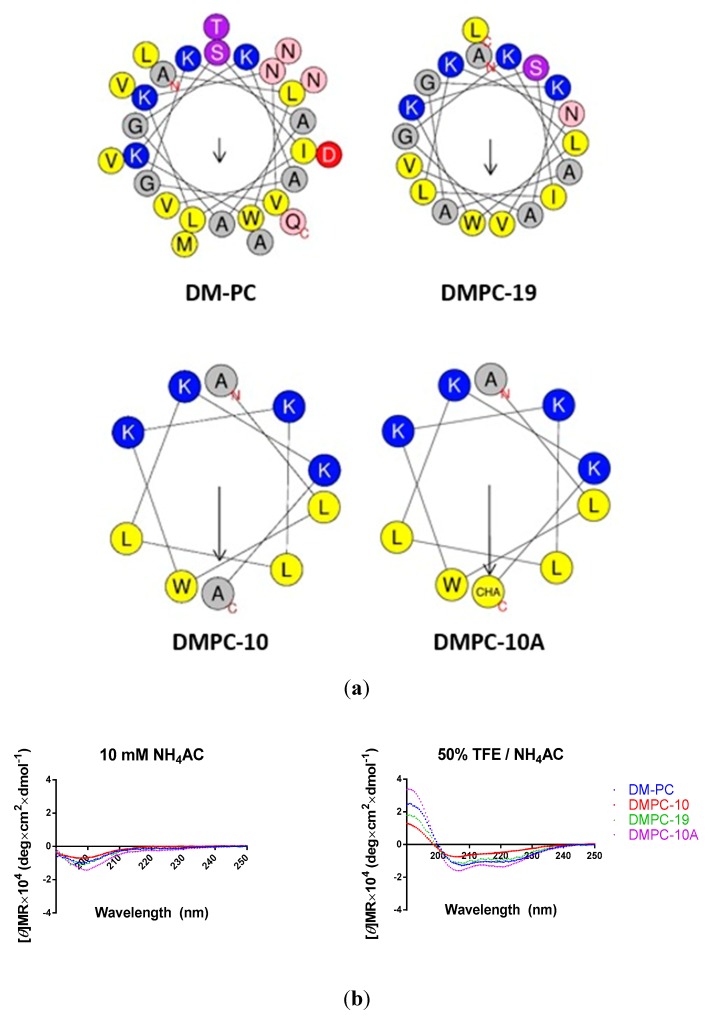
Secondary structure analysis of DM-PC and the derivatives. (**a**) Helical wheel projections of the four peptides DM-PC, DMPC-19, DMPC-10 and DMPC-10A with arrows indicating the direction of summed vectors of hydrophobicity. (**b**) CD spectra recorded for the four peptides (100 μM) in 10mM in ammonium acetate buffer and in 50% TFE ammonium acetate buffer.

Dermaseptin peptides have previously been shown to form helical structures at the N-terminal domain, and truncated N-terminal derivatives have been shown to demonstrate slightly reduced antimicrobial activity, but a marked decrease in their cytotoxicity [15,16]. However, this study only focused on the 16-mer and 13-mer truncated N-terminal derivatives. Therefore, we designed a 19-mer truncated N-terminal derivative (DMPC-19) which retained the C-terminal amide to further increase the net positive charge (Table 1) and showed an intact hydrophobic face according to helical wheel projections (Figure 4a).

The 10-mer derivative from dermaseptin S4 exhibited negligible biological potency and it was speculated this was due to the loss of hydrophobicity and positive charge [16]. It is widely accepted that a sufficient positive charge is vital for peptides to attach to bacterial surfaces via electrostatic bonding, usually +4 to +6, in which the electrostatic adsorption of AMPs to the negatively charged bacterial membrane surface can be achieved [4,22,23]. To further examine this, we selected the N-terminal decapeptide of DM-PC, ALWKSILKNV, for further design. However, two polar amino acid residues, Ser and Asp, were replaced by Lys in order to provide a sufficient positive charge. Moreover, Ile was replaced by Leu, based on the sequence of 10-mer dermaseptin S4 derivative (ALWKTLLKKV) in pervious study [24], and also the report showed that the antimicrobial potency of a Leu-substituted analogue was slightly enhanced compared to its Ile analogue [25]. To further improve the C-terminal hydrophobicity of the artificial decapeptide, the unnatural amino acid beta-cyclohexyl-L-alanine (Cha) was employed due to the large aliphatic side chain. To sum up, a decapeptide DMPC-10A (Table 1) was designed, and DMPC-10 whose last amino acid residue is Ala instead of Cha was synthesised to determine how Cha impacts upon bioactivity.

All synthesised peptides demonstrated random coil structures in aqueous solution but folded to helical structures in the environment presented by 50% TFE buffer solution (Figure 4b).

### 3.3. Antimicrobial Activities of DM-PC and the Derivatives

As shown in Table 2, DM-PC, DMPC-19 and DMPC-10A demonstrated broad-spectrum inhibitory activity against an array of microorganisms. Compared to DMPC-10, DMPC-10A showed a marked decrease in antimicrobial activity against the panel of reference and clinically isolated Gram-positive and Gram-negative bacteria, except *E. coli*, where the MIC was the same, although the MBC did increase.

Overall, the antimicrobial activity of AMPs can be affected by the concentration of cations, especially divalent cations (e.g., Ca^2+^ and Ma^2+^), which could compete with AMPs to bind to the negatively charged bacteria cells. Additionally, the environment of CF lung has high concentration of cations [26]. Therefore, we employed *P. aeruginosa* as a model to investigate the antimicrobial activity of DM-PC and derivatives in the presence of divalent cations (Table 3). With increasing concentrations of Mg^2+^, the activity of all peptides was decreased, although DMPC-10A was more resistant to these effects than the others.

### 3.4. Killing Kinetics Against P. aeruginosa

**Figure 5:**
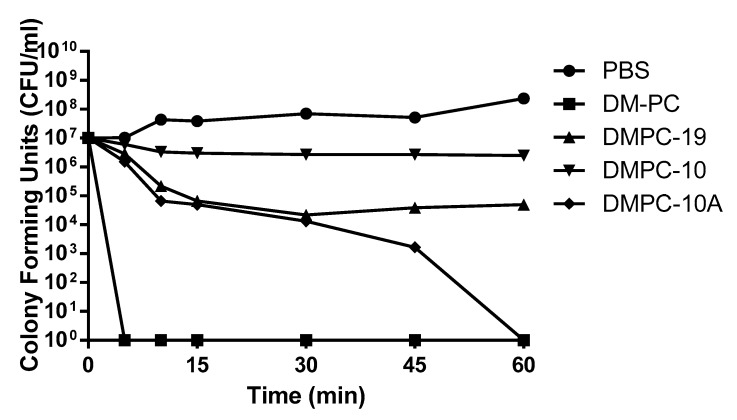
Killing effect of DM-PC and the derivatives at 8 µM against *P. aeruginosa* at various time intervals.

In a time killing assay, DM-PC and DMPC-10A revealed a potent bactericidal effect on *P. aeruginosa* (B004 V2S2 B) at 8 µM. As depicted in Figure 5, there was a noteworthy and irreversible decrease of colony forming units after five minutes exposure to DM-PC. DMPC-10A drastically reduced the colony forming units to half in 45 min, followed by complete killing after 60 min. However, although DMPC-19 inhibited the growth of bacteria at 8 µM, it failed to eradicate them.

### 3.5. Membrane Permeability Kinetics on P. aeruginosa

**Figure 6:**
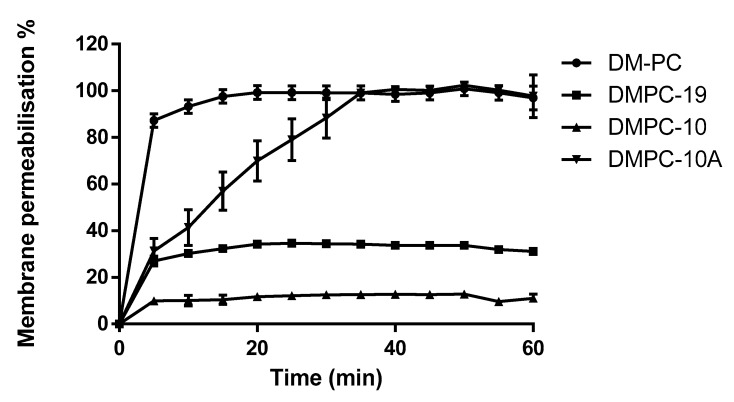
The membrane permeability of DM-PC and the derivatives at 8 µM. The percentage of membrane permeability was measured after induction by monitoring the fluorescence of SYTOX green. The error bar represents the standard error of three repeats.

The outer membrane of *P. aeruginosa* (B004 V2S2 B), whose permeability is 12–100 times less than that of *E. coli*, is a selective barrier to prevent the uptake of antibiotics [27]. Therefore, investigations on the cell membrane permeabilisation of these peptides against *P. aeruginosa* was examined (Figure 6). DM-PC and DMPC-10A showed a significant membrane permeabilization effect against *P. aeruginosa*, however, the other two derivatives exhibited much weaker activity.

### 3.6. Anti-Biofilm Activity of DM-PC and Its Derivatives

Herein, we employed MRSA and *P. aeruginosa* isolated from CF patients as models for studying the anti-biofilm activity of DM-PC and its derivatives. DM-PC, DMPC-19 and DMPC-10A effectively inhibited the formation of biofilms, as well as eradicating mature biofilms (Table 4). However, DMPC-10 exhibited negligible effects on biofilms of MRSA and *P. aeruginosa.*

### 3.7. Haemolytic Activities

**Figure 7:**
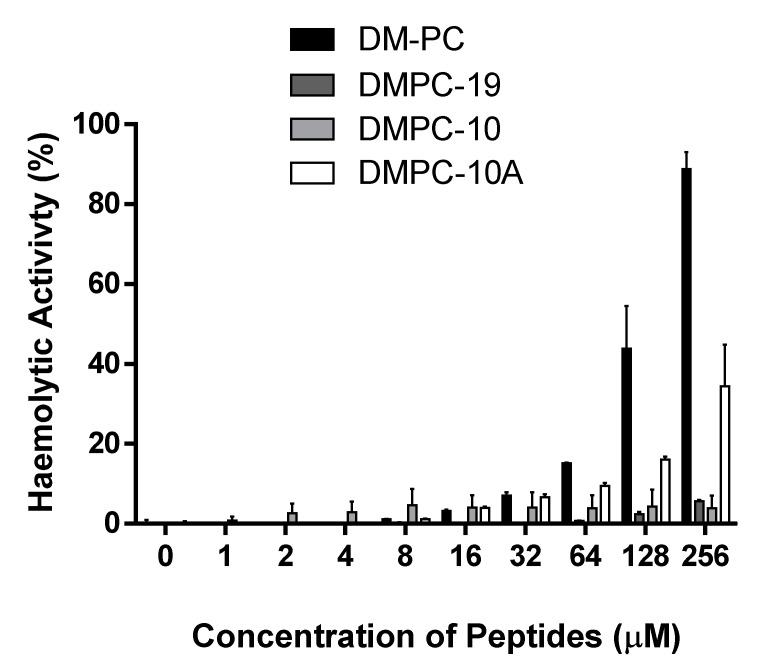
The haemolytic activities of DM-PC and the derivatives at concentrations ranging from 1 µM to 256 µM. The error bar represents the standard error of three repeats.

DM-PC demonstrates marked haemolytic activity against horse erythrocytes at higher concentrations (Figure 7), and as expected DMPC-19 exhibited little or no haemolytic activity until 256 µM. DMPC-10 also showed extremely low haemolytic activity at all concentrations, but DMPC-10A exhibited a significant increase in haemolytic activity.

## 4. Discussion

Host-defence peptides have emerged as highly potent alternatives to antibiotics, exerting powerful antimicrobial effects, even against multidrug-resistant bacterial strains [28,29]. However, some intrinsic weaknesses, like poor chemical and physical stability, tendency for aggregation, short half-life and fast elimination, limit the direct usage of naturally occurring peptides as clinical therapeutics [30]. Consequently, rational peptide design focused on mitigating these drawbacks is the key contributor to their future application as therapeutic antimicrobial peptides [31,32,33].

In this study, a novel dermaseptin, DM-PC was identified and shown to demonstrate broad-spectrum inhibitory effects against wild-type bacterial strains as well as clinical isolates. Nevertheless, its cytotoxicity toward erythrocytes is an obstacle to DM-PC being seen as a safe and effective therapeutic agent. Compared with our previous study, DM-PC and dermseptin-PS4 (DM-PS4) display high degree of structural similarity, but DM-PC demonstrate slightly strong antimicrobial activity [34]. Therefore, DM-PC was utilised as the template to design peptide analogues with a higher selectivity between the target microorganisms and mammalian cells. Our results revealed that the cytotoxicity of DM-PC can be reduced by shortening the peptide chain length and introducing several modifications to the primary sequence. DMPC-19, generated by the deletion of nine amino acid residues from the C-terminus, showed a marked reduction in its haemolytic activity, and still retained good antimicrobial activity, although not being as potent as the parent peptide. On the other hand, the artificial decapeptide, DMPC-10A, exhibited similar antimicrobial activity in comparison to the parent peptide DM-PC, but also demonstrated higher haemolytic activity than DMPC-19. In contrast, that the Cha resuidue at the C-terminus of DMPC-10A was substituted by Ala markedly reduced both its antimicrobial activity, and its cytotoxicity.

A previous study which investigated truncated dermaseptin S4 16-mer, 13-mer and 10-mer derivatives [15,16], indicated that dermaseptin exhibited a well-defined helical structure at the N-terminus, but rather loose structures at its C-terminus, and the truncated derivatives were still active against microorganisms, even though the charge and hydrophobicity were decreased [16,24,35,36]. Therefore, when designing the truncated derivatives from DM-PC, the net positive charge was optimised first. DM-PC contained an acidic amino acid residue at its C-terminus, and DMPC-19 possessed +5 net charge after removing the C-terminal segment. The dermaseptin S4 derivatives had already shown that a +5 net charge is essential for exerting broad-spectrum antimicrobial activity [35]. Indeed, the antimicrobial activity of DMPC-19 was only slightly altered, except against *E. faecalis,* where its activity was much less potent. The 10-mer dermaseptin S4 derivative had been ineffective, therefore we optimised the net charge to +5 and improved the hydrophobicity at the C-terminus. However, the antimicrobial activity of DMPC-10 was still very low, although when Cha, in which the benzene ring of Phe was replaced by cyclohexane, was employed at the C-terminus, the antimicrobial activity was restored. This indicates that the positive charge is not enough for shorter derivatives, such as K_4_S4(1-10) a from dermaseptin S4 [24]. But higher hydrophobicity, especially at the C-terminus, can improve the bioactivity. As Kustanovich suggested, the interaction with cell membrane of N-terminal domain mainly depends on the net charge state while the C-terminal domain also contributes to the binding affinity [24]. That the antimicrobial potency of DMPC-19 was retained after removal of C-terminus may be the compensation of increasing the net charges. However, when removing more residues from C-terminus, the bioactivity decreased even the net charge was unchanged, because of the reduction in the insertion affinity of peptides to lipid bilayer that contributed by the C-terminal hydrophobic domain [37]. It also correlates with the membrane permeability effect and haemolysis for the peptides in this study. As DM-PC and DM-PS4 show, DM-PC contains a Val at position 18, where is an Ala in DM-PS4. So, the stronger hydrophobicity produced by Val could result in the slightly enhancement of antimicrobial and haemolytic activity of DM-PC than DM-PS4. Compared to the short derivatives, DM-PC possesses the intact sequence that increases the binding affinity and insertion affinity towards lipid bilayers. DMPC-10A also exhibits disruption of the cell membranes of both red blood cells and *P. aeruginosa* but is less effective than DM-PC. This would indicate that optimisation of peptide length and the hydrophobicity of the C-terminus could be key to improving the therapeutic window for dermaseptin derivatives.

Additionally, NMR analysis showed that the helical structures were formed from the entire 16-mer and 13-mer dermaseptin S4 derivatives, but the 10-mer peptide was unable to fold in the 20% TFE environment [24]. Our data illustrated that DMPC-10 is able to form a helical structure in the 50% TFE environment. This might be explained by the difference of TFE % applied in the CD analysis. On the other hand, DMPC-10A possessed well-defined helical conformation in this study due to the presence of Cha, which has been reported to promote helices formation [38].

Because of the activity of DM-PC and its derivatives on the CF isolated MRSA and *P. aeruginosa*, these strains were used to investigate their anti-biofilm activity. Generally, the order of antibiofilm activity is DM-PC > DMPC-10A > DMPC-19. From the data, DMPC-19 and DMPC-10A demonstrated activity against biofilm formation but much less potent inhibition against mature biofilms. This could indicate these peptides are binding to the surface of the bacteria to prevent their attachment to biotic or abiotic surfaces. However, they could not eradicate the extracellular polymeric substances (EPS) matrix unless both derivatives presented at high concentrations. Additionally, DMPC-19 and DMPC-10A demonstrated similar antimicrobial effect against *P. aeruginosa* in the planktonic form, while MBIC of DMPC-19 is lower. We speculated that the LB for biofilm assays contains more cationic salt (250 mM NaCl) than MHB that reduced the antimicrobial activity because of low salt tolerance of DMPC-19. In contrast, the much more potent impact of DM-PC on biofilms, including mature biofilms, was observed, which coincidently shows similar trend to their haemolytic effect. It suggests that the nonspecific cytolytic activity might be involved in the disruption by EPS matrix layer via the “detergent-like” dissolving function for possessing antibiofilm effect.

## 5. Conclusions

In summary, the results from our studies illustrate that DM-PC-derived short cationic amphipathic α-helical peptides have great potential for the prevention and treatment of both chronic and acute infections. This study illustrates the possibility to design shorter derivatives from dermaseptin like peptides. Making shorter dermaseptin truncates may weaken the antimicrobial activity, however it can be resolved by enhancing the hydrophobicity/lipophilicity at C-terminus. The balance of activity and cytotoxicity could be manipulated by the length, net charge, and the hydrophobicity. Furthermore, the success of design shorter dermaseptin derivative, which could be helpful for chemical synthesis and potentially reduce the cost of manufacturing, would bring new insight for development of new antibiotic alternatives

## Figures and Tables

**Table 1:** Amino acid sequence and net charge of DM-PC and the derivatives.

Peptide	Primary Sequence	Net Charge
DM-PC	ALWKSILKNVGKAAGKAVLNAVTDMVNQ-NH_2_	4
DMPC-19	ALWKSILKNVGKAAGKAVL-NH_2_	5
DMPC-10	ALWKKLLKKA-NH_2_	5
DMPC-10A	ALWKKLLKK-Cha-NH_2_	5

**Table 2:** The minimum inhibitory concentrations (MICs) and minimum bactericidal concentrations (MBCs) of DM-PC and its analogues against reference microorganisms.

Sources	Strains	MICs/MBCs (µM)			
		DM-PC	DMPC-19	DMPC-10	DMPC-10A
Reference strains	*S. aureus* NCTC 10788	2/4	2/8	64/128	4/8
	*E. coli* NCTC 10418	4/16	2/16	8/32	8/8
	*C. albicans* NCYC 1467	2/8	2/16	64/128	4/8
	MRSA ATCC 12493	2/4	8/64	>256/>256	8/16
	*E. faecalis* NCTC 12697	32/32	256/>256	>256/>256	64/64
	*K. pneumoniae* ATCC 43816	8/64	32/128	>256/>256	4/64
	*P. aeruginosa* ATCC 27853	4/8	4/16	32/128	4/4
CF isolated strains	*P. aeruginosa* B004 V2S2 B	4/16	4/16	32/>256	4/8
	MRSA B038 V1S1 A	4/4	16/16	>256/>256	8/32
	MRSA B042 V2E1 A	4/4	8/16	256/>256	4/16

**Table 3:** Effect of Mg^2+^ on the antimicrobial activity of DM-PC and derivatives against *P. aeruginosa* B004 V2S2 B.

Concentration of Mg^2+^ (mM)	MICs (µM)			
	DM-PC	DMPC-19	DMPC-10	DMPC-10A
0	4	4	32	4
2	16	64	256	16
5	64	256	>256	32
10	>256	>256	>256	256

**Table 4:** Minimal biofilm inhibitory concentrations (MBICs) and minimal biofilm eradication concentrations (MBECs) of DM-PC and its analogues against the biofilm of MRSA and *P. aeruginosa* stains.

MBIC/MBEC	DM-PC	DMPC-19	DMPC-10	DMPC-10A
MRSA B038 V1S1 A	8/64	16/256	>256/>256	16/256
MRSA B042 V2E1 A	4/32	16/256	>256/>256	4/128
*P. aeruginosa* ATCC 27853	16/32	64/128	>256/>256	8/256
*P. aeruginosa* B004 V2S2 B	16/64	64/128	>256/>256	8/256
